# Validation of a Dietary Screening Tool in a Middle-Aged Appalachian Population

**DOI:** 10.3390/nu10030345

**Published:** 2018-03-12

**Authors:** Melissa Ventura Marra, Sowmyanarayanan V. Thuppal, Elizabeth J. Johnson, Regan L. Bailey

**Affiliations:** 1 Division of Animal and Nutritional Sciences, West Virginia University, Morgantown, WV 26506, USA; 2 Department of Nutrition Science, Purdue University, West Lafayette, IN 47907, USA; tvsowmy@gmail.com (S.V.T.); reganbailey@purdue.edu (R.L.B.); 3 Jean Mayer USDA Human Nutrition Research Center on Aging at Tufts University, Boston, MA 02111, USA; elizabeth.johnson@tufts.edu

**Keywords:** diet screening, diet quality, middle-aged adults

## Abstract

Proactive nutrition screening is an effective public health strategy for identifying and targeting individuals who could benefit from making dietary improvements for primary and secondary prevention of disease. The Dietary Screening Tool (DST) was developed and validated to assess nutritional risk among rural older adults. The purpose of this study was to evaluate the utility and validity of the DST to identify nutritional risk in middle-aged adults. This cross-sectional study in middle-aged adults (45–64 year olds, *n* = 87) who reside in Appalachia, examined nutritional status using an online health survey, biochemical measures, anthropometry, and three representative 24-h dietary recalls. The Healthy Eating Index (HEI) was calculated to describe overall diet quality. Adults identified by the DST with a nutrition risk had lower HEI scores (50 vs. 64, *p* < 0.001) and were much more likely to also be considered at dietary risk by the HEI (OR 11.6; 3.2–42.6) when compared to those not at risk. Those at risk had higher energy-adjusted total fat, saturated fat, and added sugar intakes and lower intakes of dietary fiber, and several micronutrients than those classified as not at risk by the DST. Similarly, the at-risk group had significantly lower serum levels of α-carotene, β-carotene, cryptoxanthin, lutein, and zeaxanthin but did not differ in retinol or methylmalonic acid compared with those not at risk. The DST is a valid tool to identify middle-aged adults with nutritional risk.

## 1. Introduction

Diet is a well-established modifiable factor directly related to risk and treatment of the major causes of death in adults, including cardiovascular disease, stroke, and diabetes [1]. Early adoption of a healthful diet is an ideal preventative strategy; however, improvements in diet even at mid-life can positively impact health [2]. This is particularly relevant as the large cohort of baby boomers enter older adulthood. This demographic transition will result in almost 20% of the US population, being comprised of individuals older than 65 by 2025 [3]. Concomitant with aging is chronic diseases; one in five midlife adults (45–64 year olds) already has two or more chronic medical conditions [4]. This cohort of middle-aged adults is beginning older adulthood with higher rates of obesity and chronic conditions than did previous generations [5]. Taken together, these trends will place significant demands on aging adults and their families, and on healthcare systems. Thus, it is important to identify modifiable factors that may help lessen the burden of chronic diseases of aging, particularly in health-disparate areas like Appalachia. Diet-related chronic conditions in West Virginia, the only state that lies 100% in the Appalachian region, are among the highest in the nation. Nearly 45% of middle-aged adults in the state are obese, higher than in any other state in this age group [6]. West Virginia also ranks first in rates of obesity, diabetes, and hypertension [6].

The primary care setting provides an ideal place to identify individuals who could benefit from dietary improvements. Obesity treatment guidelines [7] and the U.S. Preventative Services Taskforce [8] recommend that primary care providers provide or refer to a nutrition professional those patients who could benefit from diet-related counseling. Adherence to a dietary pattern like that of the U.S. Dietary Guidelines has been associated with reduced risk of cardiovascular disease and stroke [9,10]. Proactive screening is an effective public health strategy for identifying and targeting those individuals who could make dietary improvements for primary or secondary prevention of disease. Thus, inexpensive and easy-to-use screening tools to help healthcare practitioners identify individuals at nutritional risk and who could benefit from dietary counseling are needed. Current methods of comprehensive dietary assessment (i.e., multiple 24-h recalls and food frequency questionnaires) are costly, labor intensive and impractical in a clinical setting. More importantly, they are not intended for broad-based screening, which is essential for determining nutrition risk.

The dietary screening tool (DST) can be used to assess overall diet quality and categorize eating patterns into nutrition risk categories [11,12]. Furthermore, the DST component scores can be used to tailor specific nutrition advice to patients. The questionnaire asks simple food-related questions and questions about typical eating behaviors, and takes less than 10 min to complete and score; thereby, making it feasible to administer in the patient waiting room. The DST has previously been validated for use in older adults (73–94 year olds) in rural Pennsylvania using multiple 24-h dietary recalls and biomarkers of nutritional status including serum carotenoids [11,12]. The purpose of this study was to use multiple 24-h recalls in conjunction with biochemical markers of nutritional status to assess the validity of the DST among middle-aged adults.

## 2. Materials and Methods

### 2.1. Study Design and Participants

This cross-sectional study evaluated DST scores relative to several facets of nutritional status in a community-dwelling middle-aged (45–64 year olds) Appalachian population. The study consisted of three components: an online survey to collect demographic, medical and DST data; multiple 24-h dietary recalls to assess dietary intake and in-person visits to collect anthropometric measures and blood samples. In-person sessions occurred between October 2015 and January 2016. The study protocol (1507753607) was approved by West Virginia University’s Institutional Review Board. All participants provided written informed consent and received a $100 gift card upon study completion.

Participants were non-smokers recruited from two counties in north central West Virginia by word-of-mouth and community advertising for a diet and cardiovascular risk assessment study. Potential participants were excluded if they self-reported having a pacemaker or defibrillator; had been diagnosed with cancer or kidney, heart or liver disease; had surgery in the previous 6 months; were taking anti-inflammatory or anti-coagulant medications; had a major change in diet or appetite in the previous 3 months or abused alcohol or other substances. In total, 96 participants took part in the study. Nine were omitted from this analysis due to incomplete DST survey responses.

Demographic, health-related data, and the DST responses were collected by an online survey administered using REDCap (Research Electronic Data Capture) survey software [13]. Anthropometric measures were taken at the in-person visit using standardized protocols with participants fasted, lightly clothed and without shoes. All measurements were recorded in duplicate, and averages were used for analysis. Height (cm) was measured using a Seca 274 digital mobile stadiometer (Seca, Hamburg, Germany). Weight (kg) and body fat were measured using a Seca medical Bioelectrical Composition Analyzer (mBCA) 514 (Seca, Hamburg, Germany). Waist and hip circumferences were measured using a Gulick II Tape Measure. Waist circumference (cm) was taken at the iliac crest, and values > 102 cm for men and >88 cm for women were categorized as ‘at risk’ [14]. Body Mass Index (BMI) was calculated as weight (kg)/height (m^2^) and was classified using World Health Organization classifications [15].

### 2.2. Diet Assessment

Three 24-h dietary recalls (one weekend and two weekdays) were collected over the telephone by two trained researchers. Intake data, including the use of dietary supplements, were collected and analyzed using the Nutrition Data System for Research (NDSR) Software version 2015 (May 2015) developed by the Nutrition Coordinating Center (NCC) at the University of Minnesota, Minneapolis, Minnesota [16,17]. The Healthy Eating Index (HEI) score is an overall measure of diet quality. HEI-2010 scores were calculated based on 12 diet variables according to the NDSR’s unpublished guide [18]. Total scores could range from 0 to 100 with higher scores indicating greater adherence to the 2010 Dietary Guidelines for Americans [19]. The DST was developed to screen the diet, and thus mean intakes from the diet were used to validate the DST in this population. Mean dietary intakes were energy-adjusted within DST risk category because of differential energy intakes based on sex, physical activity, and body size.

### 2.3. Dietary Biomarker Measurements

A fasting venous blood draw was performed by a trained phlebotomist at the in-person sessions. Blood was collected in SST tubes, allowed to coagulate for 30 min and centrifuged at 1600× *g* for 10 min on-site. Serum was then apportioned into 1.5 mL amber light-protective microcentrifuge tubes and placed on dry ice for transport to West Virginia University where it was frozen at −80 °C until shipped for analysis. Reverse-phase high-performance liquid chromatography, HPLC, (Alliance 2695 Waters, Milford, MA, USA) with a photodiode array detector (Waters 2475, Millipore, Milford, MA, USA) was used to separate and quantify carotenoids, retinoids and tocopherols from serum extract with a semi-bore C30 carotenoid column (3 µm, 150 mm × 4.6 mm; YMC, Carotenoid) [20]. The xanthophylls (lutein, zeaxanthin, crytoxanthin) were quantified at 445 nm, the carotenes (β-carotene, α-carotene, lycopene) at 455 nm, retinol at 340 nm and tocopherols at 292 nm. The HPLC column was maintained at 16 °C. The method yields adequate separation of all-*trans*-lutein, *cis*-lutein, all-*trans*-zeaxanthin, *cis*-zeaxanthin, cryptoxanthin, β-carotene, 13-*cis*-β-carotene, all-*trans*-β-carotene, and 9-*cis-β*-carotene, as well as four geometrical isomers of lycopene (15-*cis*, 13-*cis*, 9-*cis*, and all-*trans* lycopenes). Carotenoids, retinol and tocopherols were quantified by determining peak areas in the HPLC chromatograms calibrated against known amounts of standards. Concentrations were corrected for extraction and handling losses by monitoring the recovery of the internal standards. The lower limit of detection is 0.2 pmol for carotenoids, 2.0 pmol for retinoids, and 2.7 pmol for α-tocopherol. Additionally, samples were sent to Mayo Medical Labs for methymalonic acid (MMA) determination using liquid chromatography-tandem mass spectrometry.

### 2.4. Statistical Analysis

All analyses were performed using Statistical Analysis Software Package 9.4 (SAS Institute Inc., Cary, NC, USA). Based on the DST scores, the participants were classified into 2 risk categories: “at risk” if the DST score < 60 and “not at risk or potential risk” if the DST score ≥ 60 based on the original validation study. Means and proportions were calculated for baseline demographic variables for all individuals and by risk categories. Differences in the DST groups were tested using analysis of variance and chi-square tests where appropriate. The odds of having lower diet quality (HEI < 51) were calculated between DST risk groups. Mean dietary intakes were calculated as the average of three recalls. Energy-adjusted dietary intakes were calculated to account for differences in caloric requirements secondary to sex, body size, metabolic efficiency and physical activity and were compared between DST risk groups. Biomarkers of nutritional status were calculated and compared by DST risk groups using analysis of variance. Where appropriate, non-normal data were transformed before analysis. Isomers of certain biomarkers were combined to calculate total biomarker concentrations: lutein (*cis* lutein + *trans* lutein), *β*-carotene (13-*cis*-*β*-carotene + 9-*cis*-*β*-carotene + *trans*-*β*-carotene), and lycopene (15-*cis*-lycopene + 13-*cis*-lycopene + trans-lycopene). Lutein and *trans*-zeaxanthin were combined together. For all biomarkers, mean concentrations were calculated after adjusting for sex as circulating carotenes and xanthophylls are reportedly higher in women than in men, independent of dietary intake [21]. Apart from adjusting for sex, methylmalonic acid, retinol and *β*-carotene, and α-tocopherol were adjusted for respective dietary supplement intake: vitamin B12, vitamin A, and vitamin E. Statistical significance was set at a *p*-value value < 0.05.

## 3. Results

Descriptive characteristics of the 87 participants who completed the DST are shown in Table 1 by risk group. The participants were on average 54 ± 4.7 years old. Overall, the sample was almost exclusively non-Hispanic white (97%) with at least a high school education (99%). Less than half were men (41.4%). The average BMI was 30.8 kg/m^2^ and the majority (70%) had an at-risk waist circumference. There were no significant differences between nutrition risk groups for sex, race/ethnicity, income, presence of chronic disease, or any measures of anthropometry. Participants in the at-risk group were 4 years younger on average than those not at risk (53 years versus 57 years, *P* < 0.001). More adults had scores < 60 (at risk) than ≥60 (not at risk) (64% versus 36%). Overall, more than half of the sample reported use of a dietary supplement, and the majority of those used 1 or 2 products (65%); however, prevalence of use did not differ by DST nutrition risk category.

Adults identified as at nutrition risk by the DST (<60 points) had lower overall diet quality as assessed by the HEI-2010 (50 vs. 64, *p* < 0.001) and higher overall energy intakes when compared to those not at risk or at potential risk, and had much greater odds of also being at risk as classified by the HEI (OR: 11.6, 95% CI: 3.2–42.6).

When adjusted for energy intake (Table 2), the diets of those at risk were higher in fat, saturated fat, and added sugars and lower in protein, fiber, and PUFA:SFA ratio. Several vitamins (including A, K, C, niacin, and B6) and minerals like magnesium, phosphorus, copper, selenium, and potassium, were also significantly lower among those that screened at risk when compared to those who did not. No differences were noted between the risk groups for carbohydrate, omega-3 fatty acids, vitamins D, E, riboflavin, thiamin, B12, iron, calcium, zinc, or sodium.

The at-risk group had significantly lower serum levels of α-carotene (150 vs. 202 nmol/L, *p* = 0.008), β-carotene (444 vs. 774 nmol/L, *p* < 0.001), cryptoxanthin (148 vs. 222 nmol/L, *p* = 0.017), *trans*-zeaxanthin (98 vs. 129 nmol/L, *p* = 0.013), lutein (373 vs. 576 nmol/L, *p* = 0.001), and lutein + *trans*-zeaxanthin (471 vs. 705 nmol/mL, *p* = 0.002). There was no significant difference for lycopene, α-tocopherol, γ-tocopherol, retinol or MMA (Table 3).

## 4. Discussion

This study validated the use of the DST questionnaire for assessing nutritional risk in middle-aged adults residing in West Virginia using multiple 24-h recalls and biomarkers of carotenoid intakes. Our results indicate that the DST provides a useful and valid means to identify those at risk for overall poor dietary intakes particularly as it relates to fruit and vegetable intake. The screening tool has the potential to be used in primary care to identify patients at nutritional risk who could benefit from dietary counseling for chronic disease prevention and treatment. It can also be helpful in tailoring individual food- and behavior-specific advice to patients.

The DST yielded high construct validity as it performed well with regard to measuring overall diet quality as compared to the HEI-2010 scores and was positively associated with favorable nutrient intakes estimated by the 24-h recalls. Concurrent validity was determined to be good as the DST was consistent with the biomarkers of nutritional status regarding carotenoid status. The at-risk group had significantly lower serum levels of five of the six major carotenoids in human serum (α-carotene, β-carotene, cryptoxanthin, zeaxanthin and lutein). The DST had good predictive validity as the index predicted diet quality to a high degree; it was 12 times more likely to predict diet quality based on HEI.

The DST performed well with carotenoids, an objective biomarker for fruit and vegetable intake [21], and predicted overall diet quality based on the HEI. However, it was not able to detect differences in vitamin B12 because the population had adequate vitamin B12 status; only 3 participants had low MMA levels. Serum vitamin E was adequate, as the mean serum α-tocopherol in both groups was greater than the population mean of 30,000 nmol/L [22]. In a well-nourished population, the DST could indicate differential intakes of fruits and vegetables and distinguish between those at nutrition risk from those at potential risk based on overall diet quality. It offers a low cost, easy method to predict diet quality instead of 24-h recalls. However, it is not certain if DST is a valid tool for other markers of nutritional status such as vitamin D or essential fatty acids, as other markers of nutrition were not tested.

The DST did not perform as well in this middle-aged population as it did in the older adult population in rural Pennsylvania. In this sample, only 6.9% (*n* = 6) had scores > 75 indicating ‘not at risk’ compared to 25% in the older Pennsylvania population. Older adults are known to have better diet quality overall than middle-aged adults [23], and the West Virginia population is known to have higher diet-related chronic conditions than residents of other states. Indeed, the average HEI-2010 scores from national data show that middle-aged adults have average scores between 57 (45–54 year olds) and 62 (55–64 year olds), and the mean of our West Virginia population was below the national average [23]. In older adults, those identified as ‘at-risk’ by the DST had lower intakes of fruits and vegetables than those ‘not-at-risk’ [24]. In addition to screening and individualizing nutrition intervention plans, the DST can provide a systematic way to evaluate diets for comparison across research studies and to identify factors associated with ‘at-risk’ nutritional status. Scores and indices are preferred ways to measure diet across different research studies and cohorts [9,25].

The strengths of the study are the use of objective measures of carotenoids as a marker of fruit and vegetable intake and the use multiple 24-h recalls to estimated nutrient intake, the gold standard methods for assessing self-reported dietary intakes. There are inherent limitations with self-reported intake data, including the under-reporting of energy intake, but these limitations are present across validation studies [26]. Additionally, the sample was considerably quite small and was comprised mostly of non-Hispanic white adults. While the racial and ethnic homogeneity is representative of the population in the state and the targeted counties (94% and 96% respectively) [27] and to the rural Pennsylvania population in which the DST was originally validated, generalizability to racial and ethnically diverse populations remains unknown. This convenient sample had a higher income and educational level than the West Virginia population, thus it may not represent the Appalachian population as a whole. Future validation studies are needed in participant groups across a range of education and income levels, and in racial and ethnically diverse populations. Although there are few valid biomarkers of nutrients, additional markers such as vitamin D and essential fatty acids could be used to further validate the tool in this population. The effects of health outcomes from interventions that aim to improve diet based on individual DST results are needed to further support its widespread adoption in primary care.

## 5. Conclusions

Diet is a critical component of the diseases of public health concern in midlife including obesity, hypertension, hypercholesterolemia, and diabetes. Understanding the role of diet in the etiology and management of diseases depends on accurately assessing dietary intake. Identification and treatment of individuals that are at nutrition risk in midlife may be an effective intervention strategy to lessen the burden of morbidity and mortality associated with aging. The DST is a valid tool to identify middle-aged adults at nutritional risk and to help clinicians to tailor nutrition messaging.

## Figures and Tables

**Table 1:** Demographic characteristics and medical history by risk categories in adults aged 45–64 years.

	All (*n* = 87)	At-Risk, Score < 60 (*n* = 56)	Not-at-Risk/Possible Risk, Score ≥ 60 (*n* = 31)	*P* Value ^1^
Age, years (SE)	54 (4.7)	53 (4.6)	57 (3.8)	<0.001
Sex, %				
Men	41	48	29	0.080
Women	59	52	21	
Race/Ethnicity, %				
Non-Hispanic white	97	97	97	0.932
Non-Hispanic Black	3	3	3	
Education				
High school and above, %	99	98	100	0.454
Income, % ^2^				
≤$49,999	25	22	30	0.748
$50,000–$74,999	22	22	23	
$75,000 or more	47	47	45	
Medical Diagnoses, %				
Diabetes	7	9	3	0.517
Pre-diabetes	6	10	0	0.123
Hypertension	30	32	26	0.711
High cholesterol	49	48	52	0.313
High triglycerides	28	30	23	0.632
Supplement users, %	59	52	71	0.082
Body Composition				
Waist Circumference, cm (SE)	103 (2)	104 (2)	102 (3)	0.566
% at risk waist circumference	70	70	71	0.897
Waist Hip Ratio (SE)	0.9 (0.01)	0.9 (0.01)	0.8 (0.01)	0.080
Body Mass Index, kg/m^2^, (SE)	30.8 (0.8)	30.6 (0.8)	31.2 (2)	0.724
% Body Fat (SE)	38 (0.9)	37 (1)	41 (2)	0.090

^1^ Comparison by risk categories—ANOVA was performed for comparing continuous variables, and chi-square tests were performed for categorical variables (*p* < 0.05). ^2^ Percentages that do not add to 100 are due to missing data.

**Table 2:** Energy-adjusted mean dietary intakes estimated from multiple 24-h recalls by risk categories for 87 middle-aged adults residing in West Virginia.

	All (*n* = 87)	At-Risk, Score < 60 (*n* = 56)	Not-at-Risk/Possible Risk, Score ≥ 60 (*n* = 31)	*P* Value
	Mean (SE)			
Dietary Screening Tool Score	55.0 (1.3)	47.3 (1.0)	68.1 (1.1)	<0.001
Healthy Eating Index	54.7 (1.5)	49.5 (1.6)	64.2 (2.4)	<0.001
Energy (kcal)	1897.1 (64.3)	2032.0 (75.7)	1653.4 (105.7)	<0.001
	Energy-adjusted Mean (SE) ^1^			*P* value ^2^
Protein (g)	43.7 (1.3)	39.6 (1.2)	51.2 (0.9)	<0.001
Carbohydrate (g)	118.9 (2.1)	118.8 (2.4)	119.2 (3.8)	0.936
Fat (g)	39.7 (0.6)	41.6 (0.7)	36.2 (0.9)	<0.001
Saturated fat (g)	13.1 (0.3)	14.1 (0.3)	10.9 (0.5)	<0.001
Omega 3 fatty acid (g)	0.91 (0.03)	0.88 (0.04)	0.98 (0.07)	0.175
PUFA:SFA ratio ^3^	0.46 (0.02)	0.38 (0.03)	0.62 (0.05)	0.001
Fiber (g)	10.5 (0.4)	9.5 (0.3)	12.4 (0.9)	0.003
Added sugars (g)	26.2 (1.6)	29.2 (2.1)	20.5 (2.2)	0.007
Vitamins				
Vitamin A (RAE)	369.5 (20.7)	314.1 (19.2)	470.0 (42.1)	0.001
Vitamin D (mcg)	2.3 (0.2)	2.1 (0.1)	2.7 (0.4)	0.217
Vitamin E (mg)	4.9 (0.3)	4.5 (0.2)	5.8 (0.6)	0.053
Vitamin K (mcg)	69.8 (10.4)	44.0 (3.7)	116.5 (26.7)	0.010
Vitamin C (mg)	39.9 (3.2)	32.7 (2.9)	53.0 (6.6)	0.007
Thiamin (mg)	0.94 (0.03)	0.91 (0.02)	1.0 (0.06)	0.205
Riboflavin (mg)	1.2 (0.04)	1.1 (0.04)	1.3 (0.08)	0.070
Niacin (mg)	13.5 (0.4)	12.5 (0.4)	15.2 (0.9)	0.007
Vitamin B6 (mg)	1.04 (0.04)	0.95 (0.03)	1.21 (0.09)	0.010
Vitamin B12 (mcg)	2.4 (0.2)	2.2 (0.1)	2.8 (0.4)	0.113
Minerals				
Calcium (mg)	518.5 (19.6)	497.8 (19.4)	556.2 (42.1)	0.213
Magnesium (mg)	160.9 (5.4)	143.8 (4.4)	192.0 (11.4)	<0.001
Phosphorus (mg)	659.8 (14.9)	613.4 (13.2)	745.0 (28.9)	<0.001
Iron (mg)	7.9 (0.3)	7.7 (0.3)	8.6 (0.7)	0.206
Zinc (mg)	6.0 (0.2)	5.8 (0.2)	6.4 (0.4)	0.085
Copper (mg)	0.6 (0.02)	0.6 (0.02)	0.7 (0.04)	0.002
Selenium (mcg)	61.34 (1.6)	56.4 (1.5)	70.3 (2.9)	<0.001
Sodium (mg)	1751.0 (38.6)	1760.5 (44.6)	1734.7 (75.6)	0.759
Potassium (mg)	1351.9 (38.3)	1231.2 (34.4)	1569.9 (75.7)	<0.001

^1^ Means expressed per 1000 kcal ^2^ Statistical significance was set at *p* < 0.05. ^3^ PUFA, polyunsaturated fatty acid; SFA, saturated fatty acid.

**Table 3:** Mean (with 95% confidence interval) biomarker concentration by nutrition risk category.

Biochemical Marker	At-Risk, Score < 60 (*n* = 56)	Not-at-Risk/Possible Risk, Score ≥ 60 (*n* = 31)	*P* Value ^1^
α-carotene (nmol/L)	150 (113–187)	202 (151–252)	0.008
β-carotene (nmol/L)	444 (332–553)	774 (625–923)	<0.001
Cryptoxanthin (nmol/L)	148 (116–179)	222 (179–264)	0.017
Lycopene (nmol/L)	1668 (1446–1890)	1756 (1456–2056)	0.558
*trans*-Zeaxanthin (nmol/L)	98 (82–113)	129 (108–150)	0.013
Lutein (nmol/L)	373 (301–444)	576 (480–673)	0.001
Lutein + Zeaxanthin (nmol/L)	471 (385–556)	705 (590–821)	0.002
Retinol (nmol/L)	4485 (4104–4866)	4466 (3950–4982)	0.944
α-tocopherol (nmol/L)	52,449 (46,833–58,065)	52,360 (44,755–59,966)	0.985
γ-tocopherol (nmol/L)	11,477 (9461–11,477)	8798 (6074–11,521)	0.122
Methylmalonic acid (nmol/L)	185 (172–199)	171 (153–189)	0.166

^1^ All variables are adjusted for sex. Apart from sex, methylmalonic acid, retinol and β-carotene, and α-tocopherol are adjusted for vitamin B12, vitamin A and vitamin E supplement use.

## References

[1] Centers for Disease Control and Prevention Leading Causes of Death. https://www.cdc.gov/nchs/fastats/leading-causes-of-death.htm.

[2] Assmann K.E., Lassale C., Andreeva V.A., Jeandel C., Hercberg S., Galan P., Kesse-Guyot E. (2015). A Healthy Dietary Pattern at Midlife, Combined with a Regulated Energy Intake, Is Related to Increased Odds for Healthy Aging. J. Nutr..

[3] Colby S., Ortman J. (2014). The Baby Boom Cohort in the United States: 2012 to 2060.

[4] Centers for Disease Control and Prevention (CDC) Multiple Chronic Conditions among Adults 45 and Over: Trends over the Past 10 Years. National Center for Health Statistics NCHS Data Brief No. 100. https://www.cdc.gov/nchs/products/databriefs/db100.htm.

[5] King D.E., Matheson E., Chirina S., Shankar A., Broman-Fulks J. (2013). The status of baby boomers’ health in the United States: The healthiest generation?. JAMA Intern. Med..

[6] West Virginia State Obesity Data, Rates and Trends—The State of Obesity. https://stateofobesity.org/states/wv.

[7] Jensen M.D., Ryan D.H., Apovian C.M., Ard J.D., Comuzzie A.G., Donato K.A., Hu F.B., Hubbard V.S., Jakicic J.M., Kushner R.F. (2014). 2013 AHA/ACC/TOS Guideline for the Management of Overweight and Obesity in Adults: A Report of the American College of Cardiology/American Heart Association Task Force on Practice Guidelines and The Obesity Society. Circulation.

[8] LeFevre M.L., U.S. Preventive Services Task Force (2014). Behavioral counseling to promote a healthful diet and physical activity for cardiovascular disease prevention in adults with cardiovascular risk factors: U.S. Preventive Services Task Force Recommendation Statement. Ann. Intern. Med..

[9] Reedy J., Krebs-Smith S.M., Miller P.E., Liese A.D., Kahle L.L., Park Y., Subar A.F. (2014). Higher diet quality is associated with decreased risk of all-cause, cardiovascular disease, and cancer mortality among older adults. J. Nutr..

[10] Nutrition Evidence Library—Dietary Patterns Systematic Review Project|Center for Nutrition Policy and Promotion. https://www.cnpp.usda.gov/nutrition-evidence-library-dietary-patterns-systematic-review-project.

[11] Bailey R.L., Miller P.E., Mitchell D.C., Hartman T.J., Lawrence F.R., Sempos C.T., Smiciklas-Wright H. (2009). Dietary screening tool identifies nutritional risk in older adults. Am. J. Clin. Nutr..

[12] Bailey R.L., Mitchell D.C., Miller C.K., Still C.D., Jensen G.L., Tucker K.L., Smiciklas-Wright H. (2007). A dietary screening questionnaire identifies dietary patterns in older adults. J. Nutr..

[13] Harris P.A., Taylor R., Thielke R., Payne J., Gonzalez N., Conde J.G. (2009). Research electronic data capture (REDCap)—A metadata-driven methodology and workflow process for providing translational research informatics support. J. Biomed. Inform..

[14] Obesity Education Initiative Electronic Textbook—Treatment Guidelines. https://www.nhlbi.nih.gov/health-pro/guidelines/current/obesity-guidelines/e_textbook/txgd/4142.htm.

[15] World Health Organization (WHO) Physical Status: The Use and Interpretation of Anthropometry. http://www.who.int/childgrowth/publications/physical_status/en/.

[16] Schakel S.F., Buzzard I.M., Gebhardt S.E. (1997). Procedures for Estimating Nutrient Values for Food Composition Databases. J. Food Compos. Anal..

[17] Schakel S.F., Sievert Y.A., Buzzard I.M. (1988). Sources of data for developing and maintaining a nutrient database. J. Am. Diet. Assoc..

[18] Nutrition Coordinating Center (NCC), University of Minnesota Guide to Creating Variables, Needed to Calculate Scores for Each Component of the Healthy Eating Index-2015 (HEI-2015). http://www.ncc.umn.edu/healthy-eating-index-hei/.

[19] Guenther P.M., Kirkpatrick S.I., Reedy J., Krebs-Smith S.M., Buckman D.W., Dodd K.W., Casavale K.O., Carroll R.J. (2014). The Healthy Eating Index-2010 Is a Valid and Reliable Measure of Diet Quality According to the 2010 Dietary Guidelines for Americans123. J. Nutr..

[20] Johnson E.J., Neuringer M., Russell R.M., Schalch W., Snodderly D.M. (2005). Nutritional manipulation of primate retinas, III: Effects of lutein or zeaxanthin supplementation on adipose tissue and retina of xanthophyll-free monkeys. Investig. Ophthalmol. Vis. Sci..

[21] Institute of Medicine Panel on Dietary Antioxidants Related Compounds (2000). Dietary Reference Intakes for Vitamin C, Vitamin E, Selenium, and Carotenoids, in Dietary Reference Intakes for Vitamin C, Vitamin E, Selenium, and Carotenoids.

[22] Ford E.S., Schleicher R.L., Mokdad A.H., Ajani U.A., Liu S. (2006). Distribution of serum concentrations of α-tocopherol and γ-tocopherol in the US population. Am. J. Clin. Nutr..

[23] Hiza H.A.B., Casavale K.O., Guenther P.M., Davis C.A. (2013). Diet quality of Americans differs by age, sex, race/ethnicity, income, and education level. J. Acad. Nutr. Diet..

[24] Greene G.W., Lofgren I., Paulin C., Greaney M.L., Clark P.G. (2018). Differences in Psychosocial and Behavioral Variables by Dietary Screening Tool Risk Category in Older Adults. J. Acad. Nutr. Diet..

[25] Krebs-Smith S.M., Subar A.F., Reedy J. (2015). Examining Dietary Patterns in Relation to Chronic Disease: Matching Measures and Methods to Questions of Interest. Circulation.

[26] Subar A.F., Freedman L.S., Tooze J.A., Kirkpatrick S.I., Boushey C., Neuhouser M.L., Thompson F.E., Potischman N., Guenther P.M., Tarasuk V. (2015). Addressing Current Criticism Regarding the Value of Self-Report Dietary Data. J. Nutr..

[27] U.S. Census Bureau QuickFacts: West Virginia. https://www.census.gov/quickfacts/WV.

